# Identification and expression analysis of chemosensory receptors in the tarsi of fall armyworm, *Spodoptera frugiperda* (J. E. Smith)

**DOI:** 10.3389/fphys.2023.1177297

**Published:** 2023-04-10

**Authors:** Jun-Feng Dong, Hai-Bo Yang, Ding-Xu Li, Hong-Qi Yu, Cai-Hong Tian

**Affiliations:** ^1^ College of Horticulture and Plant Protection, Henan University of Science and Technology, Luoyang, Henan Province, China; ^2^ Information Center of Ministry of Natural Resources, Beijing, China; ^3^ Institute of Plant Protection, Henan Academy of Agricultural Sciences, Zhengzhou, Henan Province, China

**Keywords:** tarsal transcriptome, gustatory receptor, odorant receptor, ionotropic receptor, expression profile, *Spodoptera frugiperda*

## Abstract

Chemosensation of tarsi provides moths with the ability to detect chemical signals which are important for food recognition. However, molecular mechanisms underlying the chemosensory roles of tarsi are still unknown. The fall armyworm *Spodoptera frugiperda* is a serious moth pest that can damage many plants worldwide. In the current study, we conducted transcriptome sequencing with total RNA extracted from *S. frugiperda* tarsi. Through sequence assembly and gene annotation, 23 odorant receptors 10 gustatory receptors and 10 inotropic receptors (IRs) were identified. Further phylogenetic analysis with these genes and homologs from other insect species indicated specific genes, including ORco, carbon dioxide receptors, fructose receptor, IR co-receptors, and sugar receptors were expressed in the tarsi of *S. frugiperda*. Expression profiling with RT-qPCR in different tissues of adult *S*. *frugiperda* showed that most annotated *SfruORs* and *SfruIRs* were mainly expressed in the antennae, and most *SfruGR*s were mainly expressed in the proboscises. However, *SfruOR30*, *SfruGR9*, *SfruIR60a*, *SfruIR64a*, *SfruIR75d*, and *SfruIR76b* were also highly enriched in the tarsi of *S*. *frugiperda*. Especially *SfruGR9*, the putative fructose receptor, was predominantly expressed in the tarsi, and with its levels significantly higher in the female tarsi than in the male ones. Moreover, *SfruIR60a* was also found to be expressed with higher levels in the tarsi than in other tissues. This study not only improves our insight into the tarsal chemoreception systems of *S*. *frugiperda* but also provides useful information for further functional studies of chemosensory receptors in *S. frugiperda* tarsi.

## Introduction

Daily living and reproductive behaviors of insects, such as feeding, mating, host-seeking, oviposition, and avoiding predators, rely on the ability of chemoreception (Dahanukar et al., 2005). Chemoreception, mainly includes olfaction and gustation, refers to the sensing of chemical cues from the external environment. Of which, olfaction means the detection of airborne chemical molecules, such as host plant volatiles and sex pheromones (Carey and Carlson, 2011; Zhang et al., 2014; He et al., 2022), whereas gustation refers to the detection of contact chemicals, such as amino acids, bitter and sweet tastants (Chapman, 2003; Depetris-Chauvin et al., 2015).

When the insect has landed on plants, it subsequently taps the leaves with their tarsi, detects the chemicals on the surface of the plants, and thus directs its food seeking behaviors. Insect tarsal chemosensilla are proposed to be involved in this process through sensing of food chemicals (Blaney and Simmonds, 1990; Maher and Thiery, 2004; Gaaboub et al., 2005). The chemoreceptive function of tarsal chemosensilla in detecting various food chemicals had been demonstrated in many noctuid species. For example, specific chemosensilla on the tarsi of autumn gum moth *Mnesampela privata* had been found to be sensitive to salts, sugars, and amino acids (Calas et al., 2009). The fifth tarsomere of the forelegs of female *Helicoverpa armigera* possesses 14 gustatory chemosensilla which can be robustly provoked by sugars (i.e., maltose, sucrose, fructose, and glucose), lysine, and myo-inositol (Zhang et al., 2010).

Insect chemosensory receptors, including the odorant receptors (ORs), the gustatory receptors (GRs), and the ionotropic receptors (IRs), are expressed on the dendrite membrane of the chemosensory neurons (CSNs) that located within the chemosensilla. Chemosensory receptors could selectively recognize external ligands and are the primary determinants of the detection spectrum of chemosensilla (Lu et al., 2007; Leal, 2013; Li and Liberles, 2015). Researches on insect ORs, GRs, and IRs were all firstly launched in *Drosophila melanogaster* (Gao and Chess, 1999; Clyne et al., 2000; Benton et al., 2009). In the last 20 years, along with the great advance of molecular biological techniques, the study of chemosensory receptors has been unfolded in a variety of insect species (Benton et al., 2006; Wilson, 2013; Gadenne et al., 2016; Paoli and Galizia, 2021). Insect ORs are proposed to function as non-selective cation channels *via* heterodimerization with a subunit called ORco which is highly conserved among species (Sato, et al., 2008; Butterwick et al., 2018). Ligand spectra of OR repertoires have been studied in several species, such as *D*. *melanogaster* (Hallem and Carlson, 2006), *Anopheles gambiae* (Carey et al., 2010; Wang et al., 2010), *Spodoptera littoralis* (de Fouchier et al., 2017), and *H. armigera* (Guo et al., 2021). A great number of moth-ORs in the detection of sex pheromones have also been characterized, and these ORs were specially called pheromone receptors (PRs) (Zhang et al., 2014; Yang and Wang, 2020). Insect GRs are mainly located in taste organs, mediate the perception of non-volatile chemical cues and CO_2_ (Scott et al., 2001; Jones et al., 2007). Insect GRs can act independently or as heteromultimers, depending on the gene types (Sato et al., 2011; Xu et al., 2016). Insect IRs constitute an evolutionary distinct family of chemosensory receptors. Members in this family are three transmembrane proteins homologous to ionotropic glutamate receptors but have a divergent ligand-binding domain (Benton et al., 2009). Insect IRs can be mainly divided into two classes. “Antennal IRs” are usually expressed in the antennae and are relatively conserved among species. “Divergent IRs” appear to exist in different tissues, and their types are variable among species (Croset et al., 2010). Insect IRs are supposed to function as ion channels, and form heterodimers with one or more conserved co-receptors (i.e., IR8a, IR25a, IR76b, and IR93a) (Abuin et al., 2011; Silbering et al., 2011). IRs are demonstrated to be involved in food detection (Koh et al., 2014; Ganguly et al., 2017), acid or amine sensing (Ai et al., 2013; Hussain et al., 2018), and courtship promotion (Grosjean et al., 2011).

The function of specific chemosensory receptors expressed in the tarsal chemosensilla had been investigated in several insect species. For example, 28 GRs were identified in the tarsal sensilla of *D*. *melanogaster* and demonstrated to be involved in the recognition of sweet or bitter compounds (Ling et al., 2014). *In vitro* and *in vivo* approaches showed that PxutGR1 in tarsal sensilla responds specifically to the stimulant synephrine and represents a key receptor in host specialization in the butterfly *Papilio xuthus* (Ozaki et al., 2011). Combing ectopia expression as well as behavioral and electrophysiological investigation, PrapGr28 that shows high expression in the female tarsi of *Pieris rapae* is demonstrated to be the receptor that detects the bitter compound sinigrin (Yang et al., 2021). However, systematic identification of chemosensory receptors in the tarsi of Lepidoptera is relatively limited. In a recent study, 18 ORs, 9 GRs, and 7 IRs were annotated in the tarsal transcriptome of the Noctuidae species *Helicoverpa zea* (Dou et al., 2019). To provide more useful information for further research on molecular mechanisms underlying the food detection in moths, analyses of chemosensory receptors in the tarsi of more species are still needed.

*Spodoptera frugiperda* (J.E. Smith) (Lepidoptera: Noctuidae) is an important agricultural pest of many crops, such as maize, rice, cotton, sorghum, and sugar cane (Sparks, 1979; Meagher and Nagoshi, 2012). It is pandemic throughout the North and South America and making a significant threat in sub-Saharan Africa (Nagoshi et al., 2018) and Asia (Wu et al., 2019). In behavioural assays, adult *S. frugiperda* females of which tarsi stimulated with a range of sugars, amino acids, allelochemics, and plant extracts showed proboscis extension reflexes. Further electrophysiological recordings from tarsal chemosensilla identified different neurons responsive to sugars, amino acids, and allelochemicals. And three neurons were found to be responsive to the plant extracts (Blaney and Simmonds, 1990). However, chemosensory mechanisms concerning such electrophysiological and behavioral reactions are still unknown.

In this study, we conducted systematic transcriptome analysis of male and female *S. frugiperda* tarsi*.* A total of 43 chemoreceptor genes, including 23 ORs, 10 GRs, and 10 IRs were identified. Phylogenetic relationships of the candidate genes with chemoreceptors from other insect species were then analyzed to infer the putative functions of these genes. Finally, expression profiles of the candidate genes in different chemosensory tissues of adult *S. frugiperda* were investigated. Some chemosensory receptors that were highly expressed in the tarsi were detected. These findings give us useful information for further chemosensory mechanism investigation in the tarsi of *S*. *frugiperda* as well as in the tarsi of other moth species.

## Materials and methods

### Insect rearing and tissue collection

*S*. *frugiperda* larvae were originally collected from the corn field in Shidian county, Baoshan, Yunnan Province, China, and successive generations were maintained in the laboratory under constant conditions of 27°C ± 1°C, 70%–75% relative humidity, and a 16 h: 8 h light/dark photoperiod. The larvae were fed with artificial diets (Guo et al., 2022). Adults were provided unlimited access to 10% (v/v) honey solutions.

For transcriptome analysis, tarsi of 80 three-day-old virgin male and female moths were collected separately. For RT-qPCR measurements, tissues including male and female antennae, proboscises, tarsi, and female ovipositor (together with the pheromone gland) were collected from 50 to 80 three-day-old virginal moths. All collected samples were immersed in liquid nitrogen until the total RNA extraction.

### Transcriptome sequencing, *de novo* assembly, and gene identification

Total RNA was extracted from the tarsi of *S*. *frugiperda* using Trizol reagent (Invitrogen, Carlsbad, CA, United States) according to the manufacturer’s protocol. The RNA concentration was determined with an ND-2000 spectrophotometer (Nanodrop, Wilmington, DE, United States). cDNA libraries were constructed following our previously described protocols (Sun et al., 2020), and then sequenced on an Illumina HiSeq2000 platform (Illumina, United States) at Sangon Biotech (Shanghai, China) Co., Ltd. *De novo* assembly and annotation of unigenes were performed as follows: the raw reads were firstly processed for removing the adapter sequences and low-quality bases so as to obtain the clean reads (Bolger et al., 2014). The GC-content and Q30 values were calculated to assess the sequence quality. The clean reads were then trimmed using Trimmomatic-0.30, and then merged and assembled using Trinity (. 2.4.0). The Trinity outputs were clustered by tgicl, and then capped using Cap3 to made a collection of unigenes. The unigenes were then searched (E-values < 1e-5) for homologous sequences using BLAST tool in NCBI Nr (non-redundant) database, and the COG (Clusters of Orthologous Groups of proteins), Swiss-Prot, PFAM, KEGG (Kyoto encyclopedia of genes and genomes), and GO (gene ontology) databases. The identified putative chemosensory receptor genes were manually verified by BLASTx searching against the NCBI Nr database. The open reading frames of the putative genes were predicted by translate (https://web.expasy.org/translate/). Expression levels of candidate genes in the tarsi of *S*. *frugiperda* were estimated by the TPM (transcripts per kilobase of exon per million mapped) values using RSEM (http://deweylab.github.io/RSEM/).

### Phylogenetic analysis

Phylogenetic analyses were caried out based on the amino sequences of the candidate genes and homologs of other insect species. The OR and GR sequences from *S*. *frugiperda* (this study), *Bombyx mori*, and *H. armigera*; the IR sequences from *S*. *frugiperda* (this study), *H. armigera*, and *D*. *melanogaster* were applied in the phylogenetic analysis, respectively. Detailed methods for constructing the neighbor-joining trees were similar as we previously descried (Sun et al., 2020). The amino acid sequences used for tree building are listed in Supplementary Table S1. Node support was assessed using a bootstrap procedure based on 1,000 replicates. Constructed phylogenetic trees were colored with Figtree software (v1.4.2).

### Expression profiling

Total RNA was extracted from different tissue samples of *S*. *frugiperda* using Trizol reagent according to the manufacturer’s protocols. cDNA was then synthesized with M-MLV reverse transcriptase (Promega, Madison, WI, United States), following the manufacturer’s instructions. RT-qPCR reactions were carried out on Agilent qPCR System (Mx3005P, Agilent Technologies, CA, United States). The primer sequences which were designed with Primer Premier 5.0 software are listed in Supplementary Table S2. All reactions were performed (for three times) in a total volume of 20 µL containing 10 µL SYBR Premix Ex TaqⅡ (Takara, Dalian, China), 0.4 mM of each primer, and 2.5 ng of sample cDNA under the following conditions: 1 cycle of 94°C for 30 s; 40 cycles of 94°C for 5 s, 58°C for 30 s, and 72°C for 25 s; followed by 1 cycle of 94°C for 15 s, 60°C for 1 min, 94°C for 15 s. The 2^−ΔΔCT^ method was applied to calculate the expression levels of candidate genes (Schmittgen and Livak, 2008). *β*-*actin* gene of *S*. *frugiperda* was chosen as the internal control to normalize expression levels. Amplification curves (S-shaped) and CT values (ranging from 17.2 to 18.3) for the reactions of the *β*-*actin* gene were carefully checked to make sure it is consistent across different tissues. The primer efficiency was evaluated by standard amplification curves constructed with 10-fold dilutions of cDNA samples. The efficiency percentage (90–110%) was validated within the acceptable range. All experiments were repeated three times using three independent RNA samples. Figures were made using GraphPad Prism 6 (GraphPad Software Inc., San Diego, CA). The data were analyzed by ANOVA followed by Tukey’s multiple comparison test, and all figures were made in GraphPad Prism 6 (GraphPad Software Inc., San Diego, CA, United States). The level of significance was set at *p* < 0.05.

## Results

### Transcriptome sequencing, assembly, and annotation

A total of 48.25 million and 57.31 million clean reads (mean length 141 bp) were produced from the male and female tarsi samples, respectively. The Q30 bases ratio in each sample was ≥93.23%. All clean reads from both samples were assembled and generated a total of 100,048 unigenes. According to the size distribution analysis result, 14.55% of the unigenes were ≥1,000 bp. The N50 length of the unigenes was 968 bp and the mean length of the unigenes was 670.79 bp (Table 1). All the obtained unigenes were then aligned to Nr, KEGG, Swiss-Prot, PFAM, COG, and GO databases. Base on the BLASTx results, 56.01% (56037) of the unigenes had matches in at least one database. Among the unigenes (27,918) that annotated to the Nr database, 84.34% (23,547) had best hits to sequences from lepidopteran species (Supplementary Figure S1).

**TABLE 1:** Summary of the transcriptome assembly of *S. frugiperda* tarsi.

Length range (bp)	Transcript number	Unigene number
500+	110,319	47,791
1,000+	47,791	14,562
Total number	270,658	100,048
Total length	188,796,114	67,111,425
N50 length	1,007	968
Mean length	697.54	670.79

Blast2GO was then used to classify the genes into functional categories. Among the 100,048 unigenes, 31,635 (31.62%) were assigned to at least one GO group. Among which, 30,284 were allocated to the biological process group, 17,798 to the cellular component group, and 15,597 to the molecular function group. The most enriched terms were “binding” in the molecular function, “cell” in the biological process, and “metabolic process” in the biological process (Supplementary Figure S2).

### Identification, phylogenetic analysis, and expression profiling of candidate SfruORs

Based on the Blastx results, a total of 23 candidate OR genes (*SfruORs*) were identified in the transcriptome of *S*. *frugiperda* tarsi. Twenty-one of these genes have putative full-length ORFs. Details for the 23 SfruORs, including BLASTx best hits, lengths, and sequences are listed in Table 2 and Supplementary Table S3. Among the 23 annotated SfruORs in *S*. *frugiperda* tarsi, 12 (SfruOR1/12b/17/25/27/34/38/40/49b/57/62/64, accession numbers QQ442940−QQ442951) have not been reported in previous genome or transcriptome studies of this species (Gouin et al., 2017; Qiu et al., 2020; Sun et al., 2022). To keep consistency, candidate genes in our study were numbered following the names of previously reported sequences in *S*. *frugiperda* (if possible) or following the names of the best hit homologs in other lepidopteran species.

**TABLE 2:** Candidate chemosensory receptors in the tarsi of *S. frugiperda*.

Name	ID	ORF (aa)	BLASTx best hit (GenBank accession/name/species)	Full length	Identity (%)	E-value
ORs						
SfruOR1	DN36107_c2_g3	449	QYF65492.1| odorant receptor 1 [*Spodoptera litura*]	Yes	95	0.0
SfruOR12b	DN36647_c0_g1	388	YF65502.1| odorant receptor 12 [*Spodoptera litura*]	No	94	0.0
SfruOR17	DN33677_c0_g2	255	QYF65507.1| odorant receptor 17 [*Spodoptera litura*]	No	95	7e-178
SfruOR25	DN39992_c0_g1	416	QNS36222.1| olfactory receptor 25 [*Mythimna separata*]	Yes	75	1e-174
SfruOR27	DN33283_c0_g1	429	QYF65517.1| odorant receptor 27 [*Spodoptera litura*]	Yes	98	0.0
SfruOR30	DN44157_c8_g3	387	XP_050551229.1| Or1-like [*Spodoptera frugiperda*]	Yes	92	0.0
SfruOR32	DN41457_c1_g1	397	QEY02574.1| odorant receptor 5 [*Spodoptera littoralis*]	Yes	72	0.0
SfruOR34	DN32907_c0_g1	409	QYF65523.1| odorant receptor 34 [*Spodoptera litura*]	Yes	93	0.0
SfruOR35	DN38262_c0_g2	453	XP_022831643.1| odorant receptor 85c-like [*Spodoptera litura*]	Yes	95	0.0
SfruOR38	DN38347_c0_g1	418	QNS36229.1| olfactory receptor 38 [*Mythimna separata*]	Yes	86	1e-164
SfruOR40	DN32294_c0_g1	412	ALM26230.1| odorant receptor 40 [*Athetis dissimilis*]	Yes	76	5e-167
SfruOR45	DN39388_c1_g1	429	XP_022825109.1| odorant receptor 13a-like isoform X1 [*Spodoptera litura*]	Yes	94	0.0
SfruOR49a	DN36640_c0_g1	403	XP_035447756.2| odorant receptor 49a-like [*Spodoptera frugiperda*]	Yes	100	0.0
SfruOR49b	DN32423_c0_g1	393	XP_022834239.1| odorant receptor 49b-like [*Spodoptera litura*]	Yes	96	0.0
SfruOR50	DN34812_c1_g1	404	QNS36220.1| olfactory receptor [*Mythimna separata*]	Yes	72	0.0
SfruOR53	DN34590_c0_g2	404	QYF65542.1| odorant receptor 53 [*Spodoptera litura*]	Yes	93	0.0
SfruOR57	DN41551_c0_g1	397	QYF65545.1| odorant receptor 57 [*Spodoptera litura*]	Yes	89	0.0
SfruOR62	DN39074_c3_g2	371	ALM26245.1| odorant receptor 62 [Athetis dissimilis]	Yes	93	0.0
SfruOR64	DN40969_c1_g1	413	AVF19676.1| putative odorant receptor [*Peridroma saucia*]	Yes	78	0.0
SfruOR67a	DN39579_c0_g1	416	XP_050562343.1| odorant receptor 67a-like [*Spodoptera frugiperda*]	Yes	99	0.0
SfruOR67c	DN32912_c2_g1	390	XP_050554729.1| odorant receptor 67c-like [*Spodoptera frugiperda*]	Yes	99	0.0
SfruOR85c	DN42554_c0_g2	393	XP_050557942.1| odorant receptor 85c-like [*Spodoptera frugiperda*]	Yes	99	1e-176
SfruORco	DN43518_c2_g2	473	AAW52583.1| putative chemosensory receptor 2 [*Spodoptera exigua*]	Yes	99	2.4e-258
**GRs**						
SfruGR1	DN33391_c0_g2	464	XP_022828173.1| gustatory and odorant receptor 22 [*Spodoptera litura*]	Yes	99	0.0
SfruGR2	DN28721_c0_g1	433	XP_035439638.1| gustatory and odorant receptor 22-like [*Spodoptera frugiperda*]	Yes	100	0.0
SfruGR3	DN30952_c0_g1	475	XP_022815658.1| gustatory and odorant receptor 24 [*Spodoptera litura*]	Yes	100	0.0
SfruGR4	DN31889_c2_g1	402	XP_035430303.1| gustatory receptor for sugar taste 64a-like [*Spodoptera frugiperda*]	Yes	100	0.0
SfruGR5	DN30906_c0_g1	476	XP_035430301.2| gustatory receptor for sugar taste 64e-like [*Spodoptera frugiperda*]	Yes	100	0.0
SfruGR6	DN34523_c0_g1	447	QYF65556.1| gustatory receptor 6 [*Spodoptera litura*]	No	95	0.0
SfruGR7	DN37736_c0_g1	429	XP_035429284.2| gustatory receptor for sugar taste 64a-like [*Spodoptera frugiperda*]	Yes	100	0.0
SfruGR8	DN33017_c0_g1	431	XP_035429285.2| gustatory receptor for sugar taste 64a-like [*Spodoptera frugiperda*]	Yes	100	0.0
SfruGR9	DN40413_c0_g1	488	XP_035448630.1| gustatory receptor for sugar taste 43a [*Spodoptera frugiperda*]	Yes	100	0.0
SfruGR10	DN47744_c0_g1	430	XP_050553314.1| gustatory receptor for sugar taste 64f-like [*Spodoptera frugiperda*]	Yes	100	0.0
**IRs**						
SfruIR8a	DN34445_c1_g1	898	QYF65596.1|ionotropic receptor 8a [*Spodoptera litura*]	Yes	96	0.0
SfruIR21a	DN30270_c2_g1	852	XP_035448875.2| ionotropic receptor 21a [*Spodoptera frugiperda*]	Yes	100	0.0
SfruIR25a	DN42711_c2_g3	918	XP_022828195.1| ionotropic receptor 25a [*Spodoptera litura*]	Yes	99	0.0
SfruIR60a	DN43581_c1_g1	660	QHB15321.1| ionotropic receptor 60a [*Peridroma saucia*]	Yes	80	0.0
SfruIR64a	DN38775_c1_g1	603	ARB05666.1| ionization receptor 64a [*Mythimna separata*]	Yes	80	0.0
SfruIR75a	DN39469_c0_g1	631	XP_035459405.2| ionotropic receptor 75a [*Spodoptera frugiperda*]	Yes	100	0.0
SfruIR75d	DN36075_c1_g1	593	ADR64683.1| chemosensory ionotropic receptor IR75d [*Spodoptera littoralis*]	Yes	95	0.0
SfruIR75p	DN38967_c0_g1	624	XP_022816386.1| glutamate receptor 1-like [*Spodoptera litura*]	Yes	90	0.0
SfruIR76b	DN42735_c2_g1	542	ADR64687.1 |putative chemosensory ionotropic receptor IR76b [*Spodoptera littoralis*]	Yes	95	0.0
SfruIR93a	DN42070_c0_g1	708	XP_050563436.1| ionotropic receptor 93a [*Spodoptera frugiperda*]	Yes	100	0.0

**FIGURE 1:**
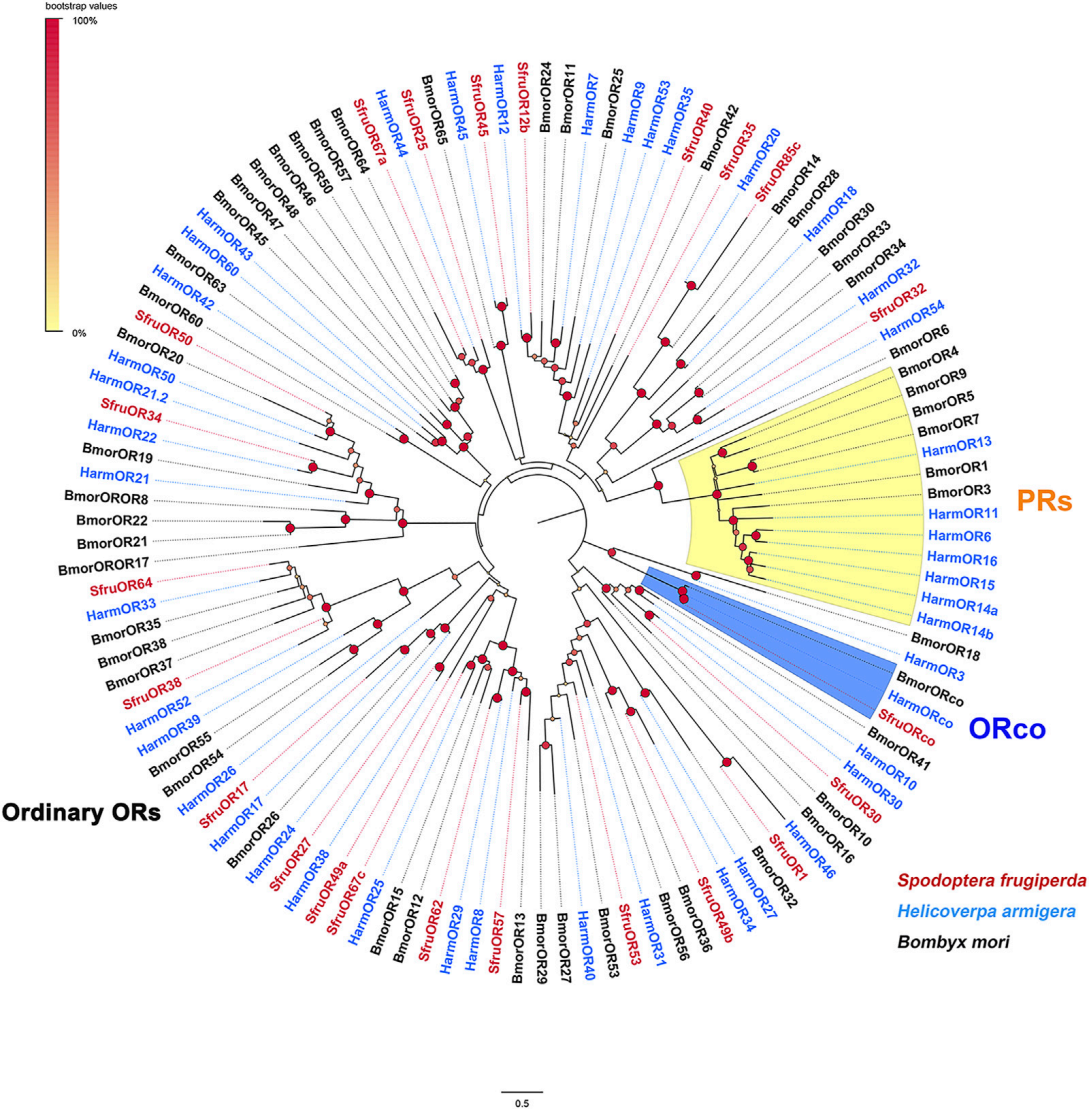
Phylogenetic analysis of the candidate SfruORs from the transcriptome of *S*. *frugiperda* tarsi and ORs from *H*. *armigera* and *B*. *mori*.

To infer the putative functions of the candidate SfruORs in this study, phylogenetic relationships were analyzed based on the alignment with ORs from *B*. *mori* and *H*. *armigera*. According to the neighbor-joining tree, ORcos were highly conserved and clustered in the ORco branch. Other 22 SfruORs were scatter in different “ordinary ORs” branches, and we did not find the putative “PR” genes in the transcriptome of *S*. *frugiperda* tarsi (Figure 1).

**FIGURE 2:**
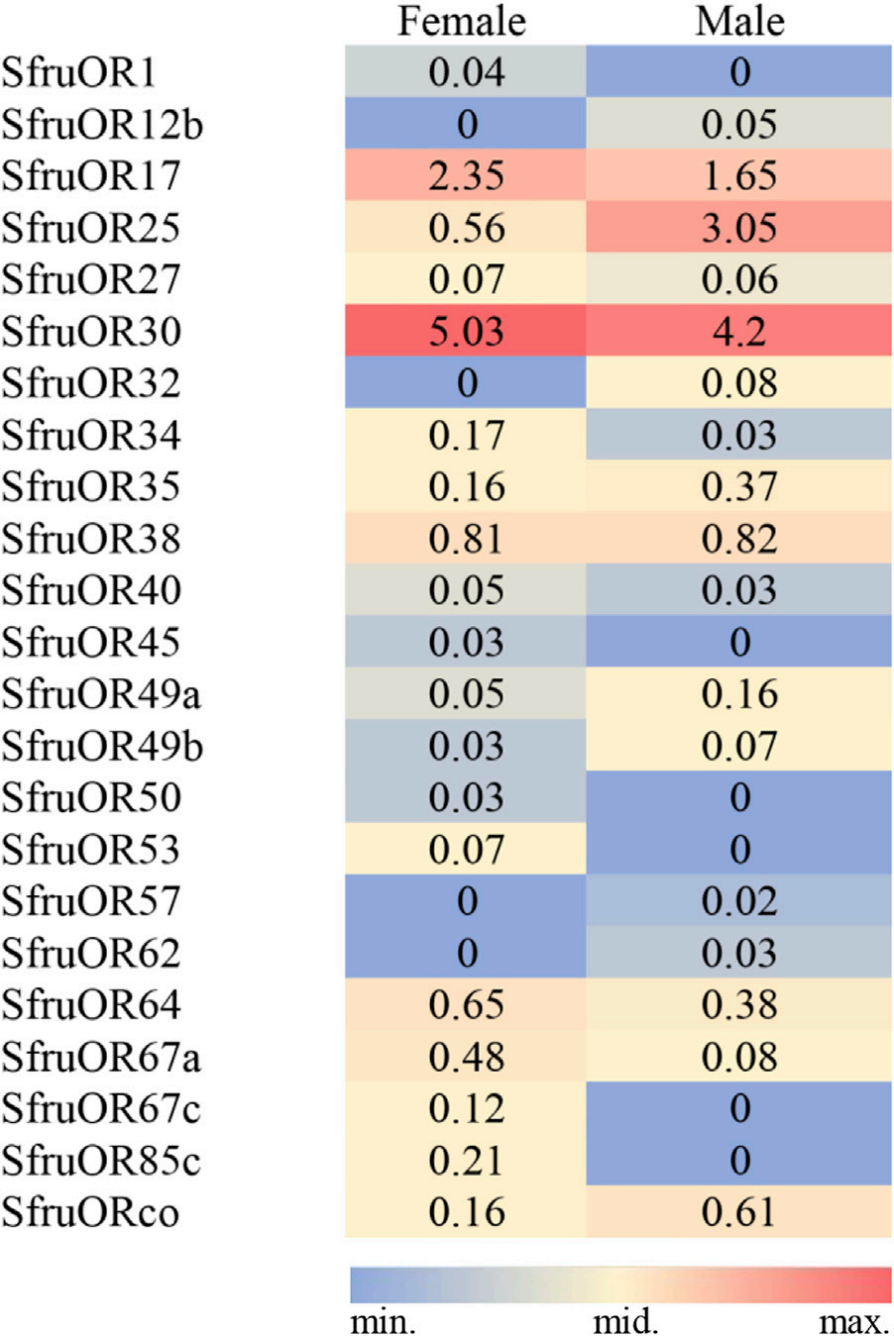
TPM values of candidate SfruORs in the tarsi of male and female *S*. *frugiperda*.

The expression of the 43 (23 *SfruORs*,10 *SfruGRs*, and 10 *SfruIRs*) candidate receptor genes was then normalized across the transcriptome sequences using the TPM calculation method. For *SfruORs*, despite the inconsistency of TPM values between females and males, *SfruOR30* was the highest transcribed gene (5.03/4.20 TPM, females/males, similarly hereinafter) among all the annotated *SfruORs* in the tarsi of *S*. *frugiperda*. Other two candidate *SfruORs*, *SfruOR17* and *SfruOR27*, showed high expression levels as indicated by the TPM values (2.35/1.65 for *SfruOR17*, 0.56/3.05 for *SfruOR27*). Significantly, transcripts of *SfruORco* were also detected in the tarsi, although its TPM values were quite low (0.16/0.61) (Figure 2).

**FIGURE 3:**
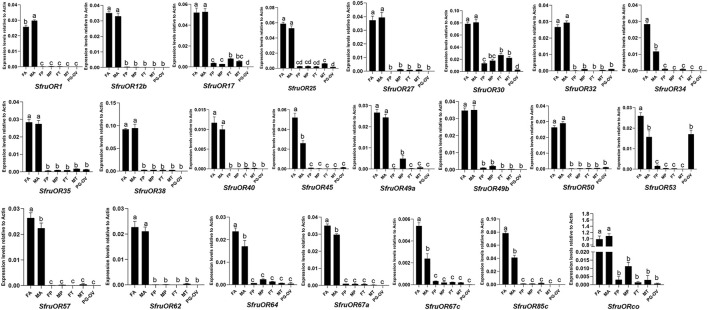
Expression patterns of candidate *SfruORs* in different chemosensory tissues of adult *S*. *frugiperda*. FA: female antennae, MA: male antennae, FP: female proboscises, MP: male proboscises, FT: female tarsi, MT: male tarsi, PG-OV: pheromone gland-ovipositor. Different letters indicate significant difference based on a one-way ANOVA followed by Tukey’s multiple comparison test. Error bars show the standard errors of the means (+SE), *p* < 0.05, n = 3.

RT-qPCR was further performed to study the expression profiles of candidate receptor genes in various chemosensory tissues of adult *S*. *frugiperda*, including the antennae, tarsi, proboscises, and ovipositors. As shown in Figure 3, all of the *SfruOR* genes were mainly expressed in the antennae, and the levels of *SfruOR34*/*45*/*53*/*57*/*64*/*67c*/*85c* were higher in female antennae than in male ones. The results also showed that *SfruOR30* was highly expressed in the tarsi (both sexes), with its levels lower than in the antennae, but higher than in other tested tissues (i.e., proboscises and ovipositors).

### Identification, phylogenetic analysis, and expression profiling of candidate SfruGRs

Ten putative SfruGRs were identified based on the analysis of the transcriptome of *S*. *frugiperda* tarsi. Except for SfruGR6, all the other annotated SfruGRs possess complete ORFs (Table 2). Among the 10 SfruGRs, SfruGR1/2/3/9 have been reported in previous larval antennae and maxilla transcriptome studies of this species (Sun et al., 2022). SfruGR4/5/6//7/8/10, which have best hits to the putative gustatory receptor for sugar taste 64a/e/f-like in genome sequences, have not yet been annotated in other transcriptome data of this species.

**FIGURE 4:**
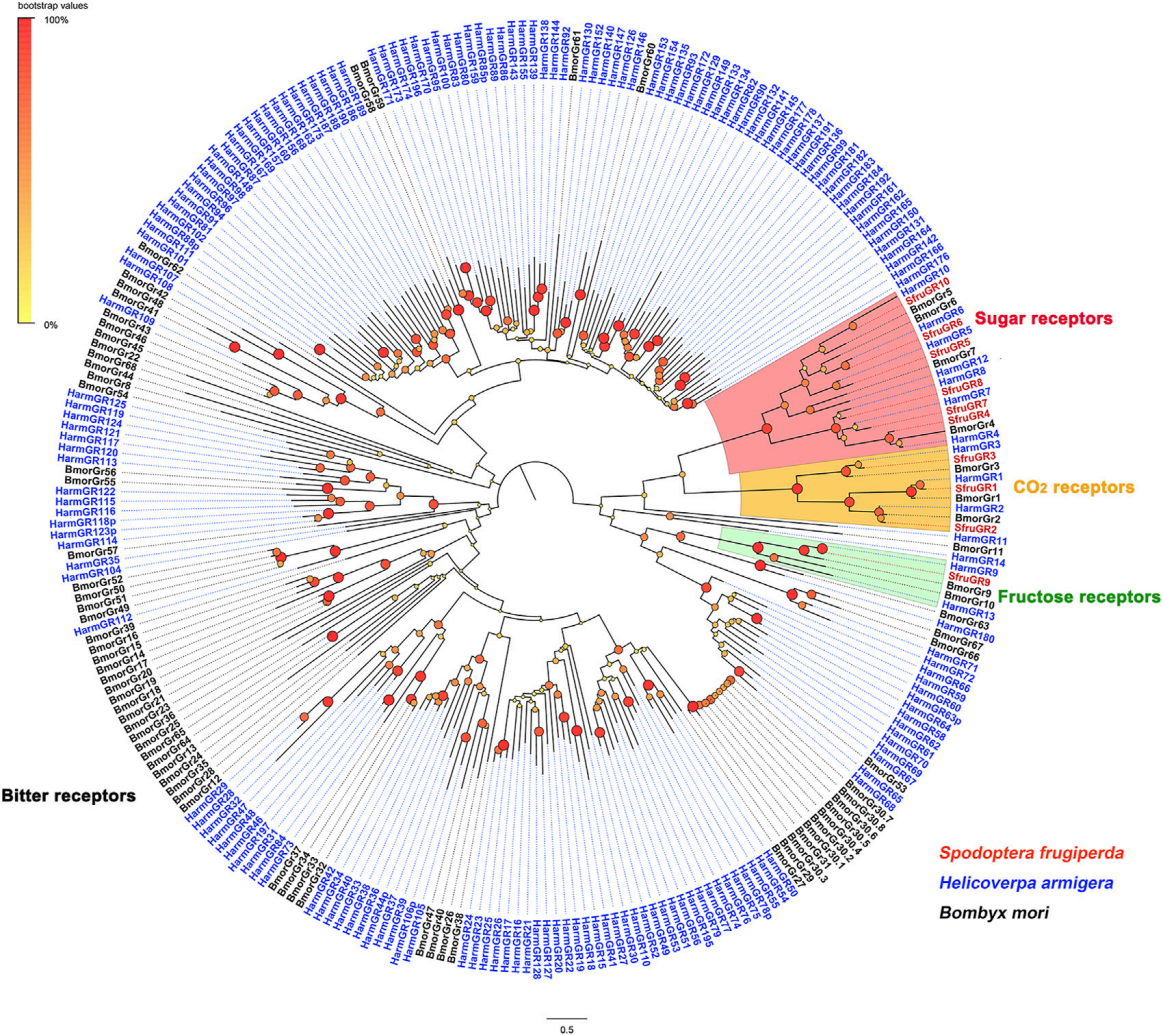
Phylogenetic analysis of the candidate SfruGRs from the transcriptome of *S*. *frugiperda* tarsi and GRs from *H*. *armigera* and *B*. *mori*.

A phylogenetic tree was then built with GRs from *S*. *frugiperda* (this study), *B*. *mori*, and *H*. *armigera*. The results showed that SfruGR1, SfruGR2, and SfruGR3, which grouped with BmorGR1/HarmGR1, BmorGR2/HarmGR2, and BmorGR3/HarmGR3 linages, were putative CO_2_ receptors. SfruGR9, clustered with the BmorGR9, was putative fructose receptor. Other six SfruGRs (SfruGR4/5/6/7/8/10) clustered in the “sugar-taste receptors” groups (Figure 4).

**FIGURE 5:**
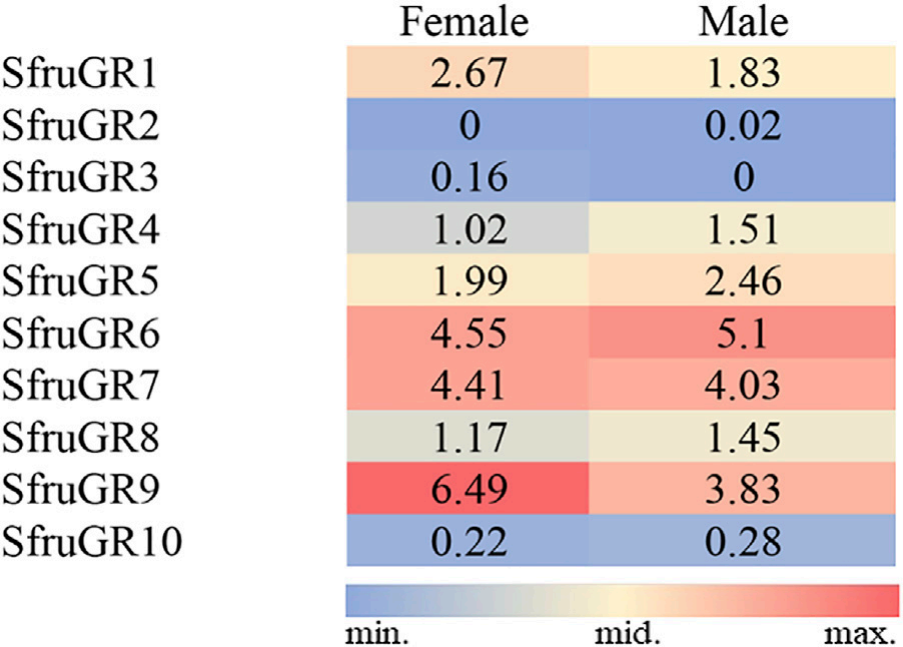
TPM values of candidate SfruGRs in the tarsi of male and female *S*. *frugiperda*.

According to the TPM values, transcript levels of *SfruGR9* in the tarsi were more abundant than that of the other annotated *SfruGRs*, and with its TPM value higher in the female tarsi (6.49 TPM) than in the male ones (3.83 TPM). Other two sugar-taste receptors, *SfruGR6* and *SfruGR7*, were also highly enriched in the tarsi, and with their transcript levels similar between females and males. In comparison, the two putative CO_2_ receptors, *SfruGR2* (0/0.02) and *SfruGR3* (0.16/0) have the lowest TPM values in the *S*. *frugiperda* tarsi (Figure 5).

**FIGURE 6:**
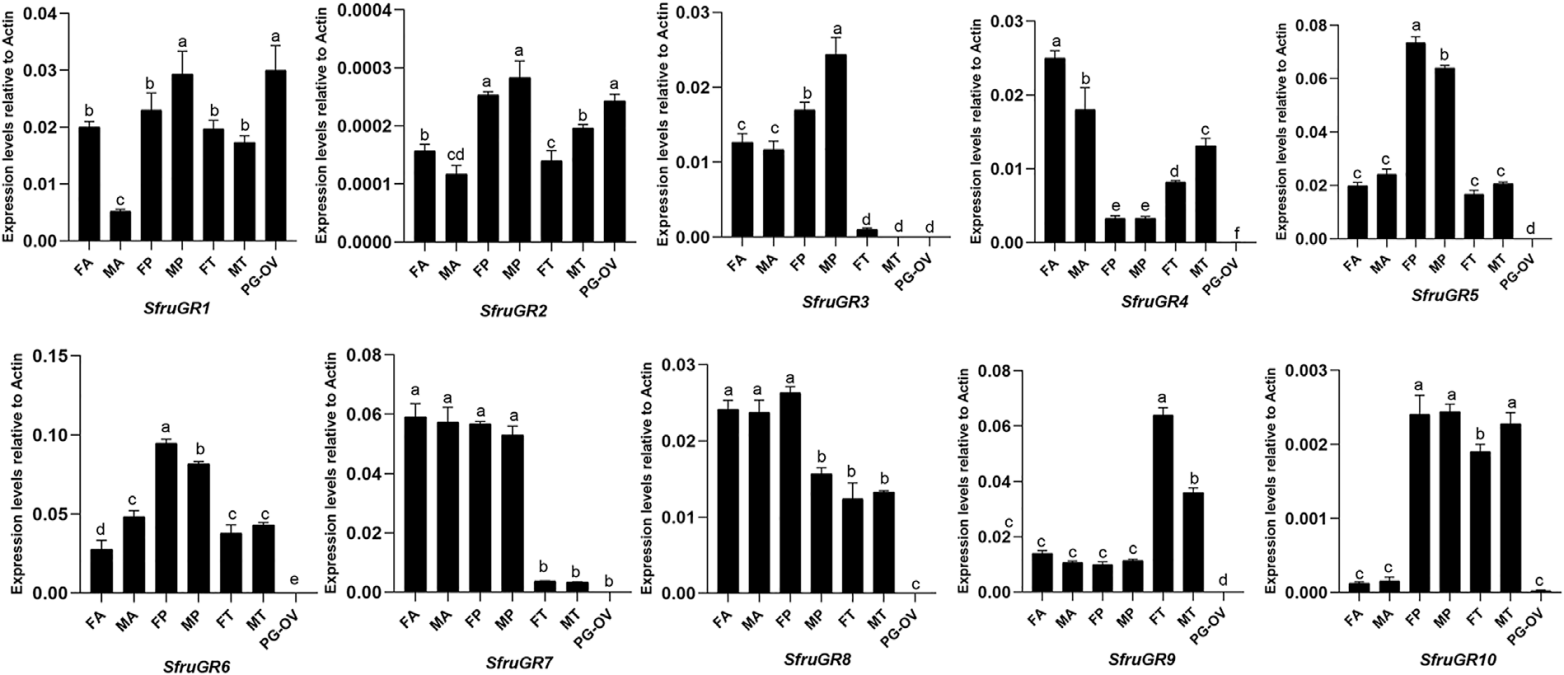
Expression patterns of candidate *SfruGRs* in different chemosensory tissues of adult *S*. *frugiperda*. FA: female antennae, MA: male antennae, FP: female proboscises, MP: male proboscises, FT: female tarsi, MT: male tarsi, PG-OV: female pheromone gland-ovipositor. Different letters indicate significant difference based on a one-way ANOVA followed by Tukey’s multiple comparison test. Error bars show the standard errors of the means (+SE), *p* < 0.05, n = 3.

Based on RT-qPCR analyses, *SfruGR9* showed significantly higher expression levels in the tarsi than in other tissues, and its expression in female tarsi was more abundant than in the male ones. This is accordant with the expression profiles of *SfruGR9* as that reported by Sun et al., 2022. Eight *SfruGR* genes, *SfruGR1*/*2*/*3*/*5*/*6*/*7*/*8*/*10*, were mainly expressed in the proboscises of both sexes, among which, *SfruGR10* was also highly expressed in the tarsi of both sexes. On contrast, *SfruGR4* was mainly expressed in the antennae, and with levels higher in the female moths than in the male ones (Figure 6).

### Identification, phylogenetic analysis, and expression profiling of candidate SfruIRs

We annotated 10 *SfruIRs* in the transcriptome of *S*. *frugiperda* tarsi. All of these candidate IRs were identified possessing complete ORFs (Table 2, Supplementary Table S3). Based on the Blastx results, sequences of 9 *SfruIRs* have been uploaded to the NCBI database previously, except for SfruIR8a (accession number OQ442939), which has not been reported in previous genome or transcriptome studies of this species (Gouin et al., 2017; Qiu et al., 2020; Sun et al., 2022).

**FIGURE 7:**
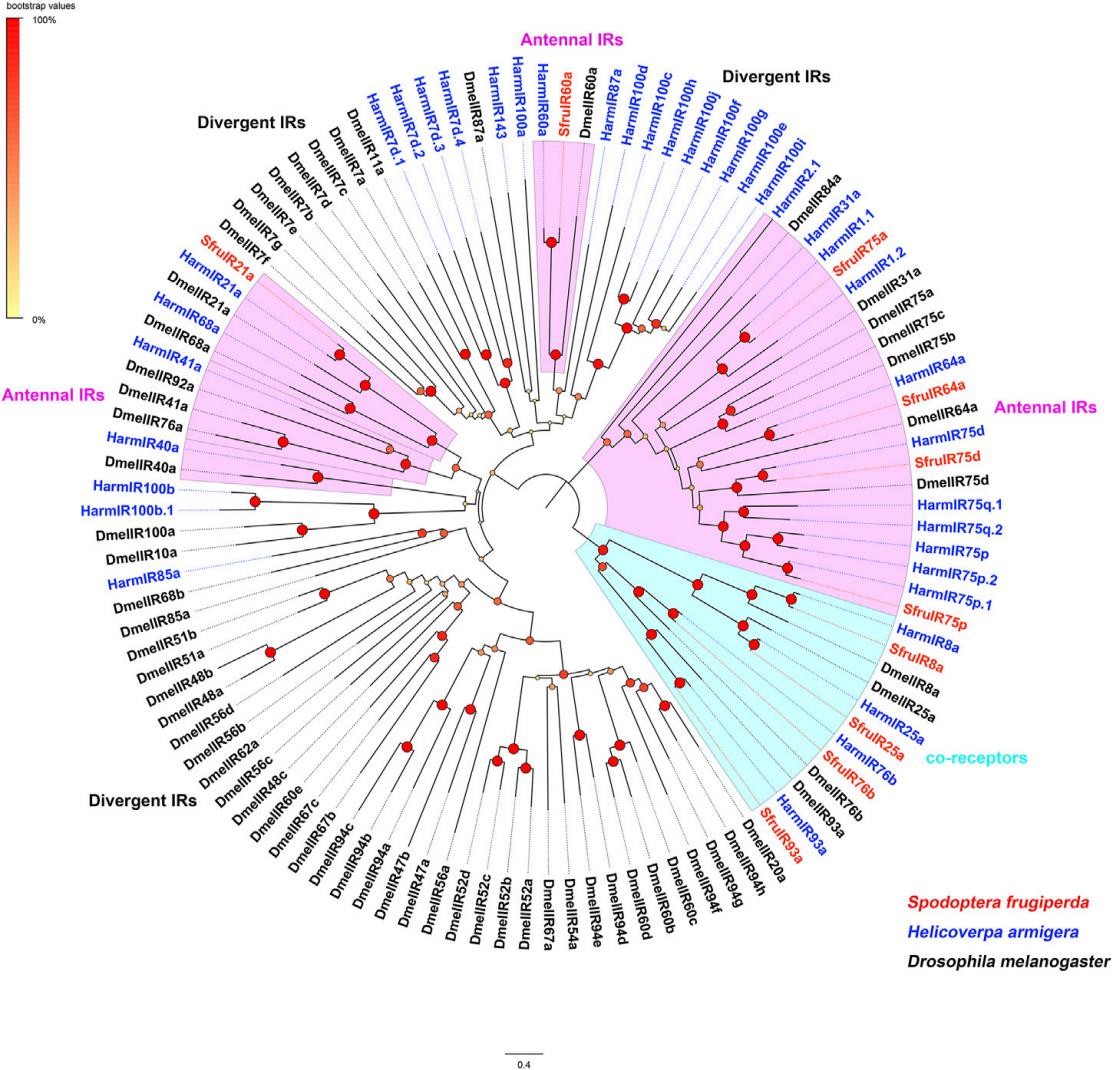
Phylogenetic analysis of the candidate SfruIRs from the transcriptome of *S*. *frugiperda* tarsi and IRs from *H*. *armigera* and *D*. *melanogaster*.

A neighbor-joining tree was then constructed to predict evolutionary relationships between SfruIRs (this study) and IRs from *D*. *melanogaster* and *H*. *armigera*. Predictably, all of the four putative SfruIR co-receptors, SfruIR8a, SfruIR25a, SfruIR76b, and SfruIR93a, clustered in the co-receptor lineages of IR8a, IR25a, IR76b, and IR93a, respectively. The other 6 SfruIRs (SfruIR21a/60a/64a/75a/75d/75p) were assigned to the “antennal IR” clade (Figure 7).

**FIGURE 8:**
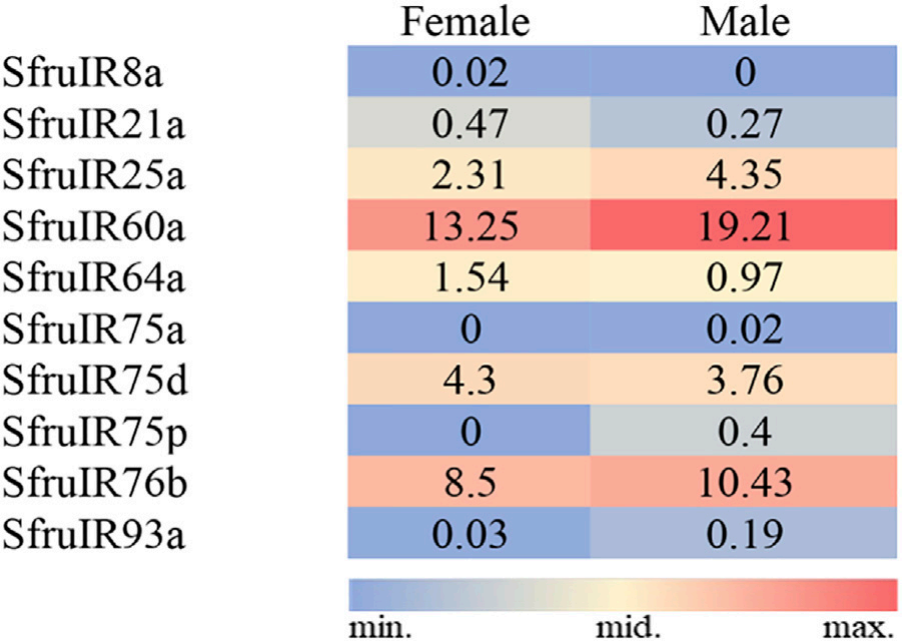
TPM values of candidate SfruIRs in the tarsi of male and female *S*. *frugiperda*.

TPM value analysis indicated that the “antennal IR”, *SfruIR60a*, had the highest transcript levels (13.25/19.21 TPM) in the *S*. *frugiperda* tarsi among all the annotated SfruIRs. Another “antennal IR”, *SfruIR75d*, was also abundantly transcribed in the tarsi (4.3/3.76 TPM). Among the 4 annotated co-receptors, *SfruIR76b* was the most enriched one in the tarsi (8.5/10.43 TPM). Other two co-receptors, *SfruIR8a* and *SfruIR93a*, showed quite low values of 0.02/0 TPM and 0.03/0.19 TPM, respectively (Figure 8).

**FIGURE 9:**
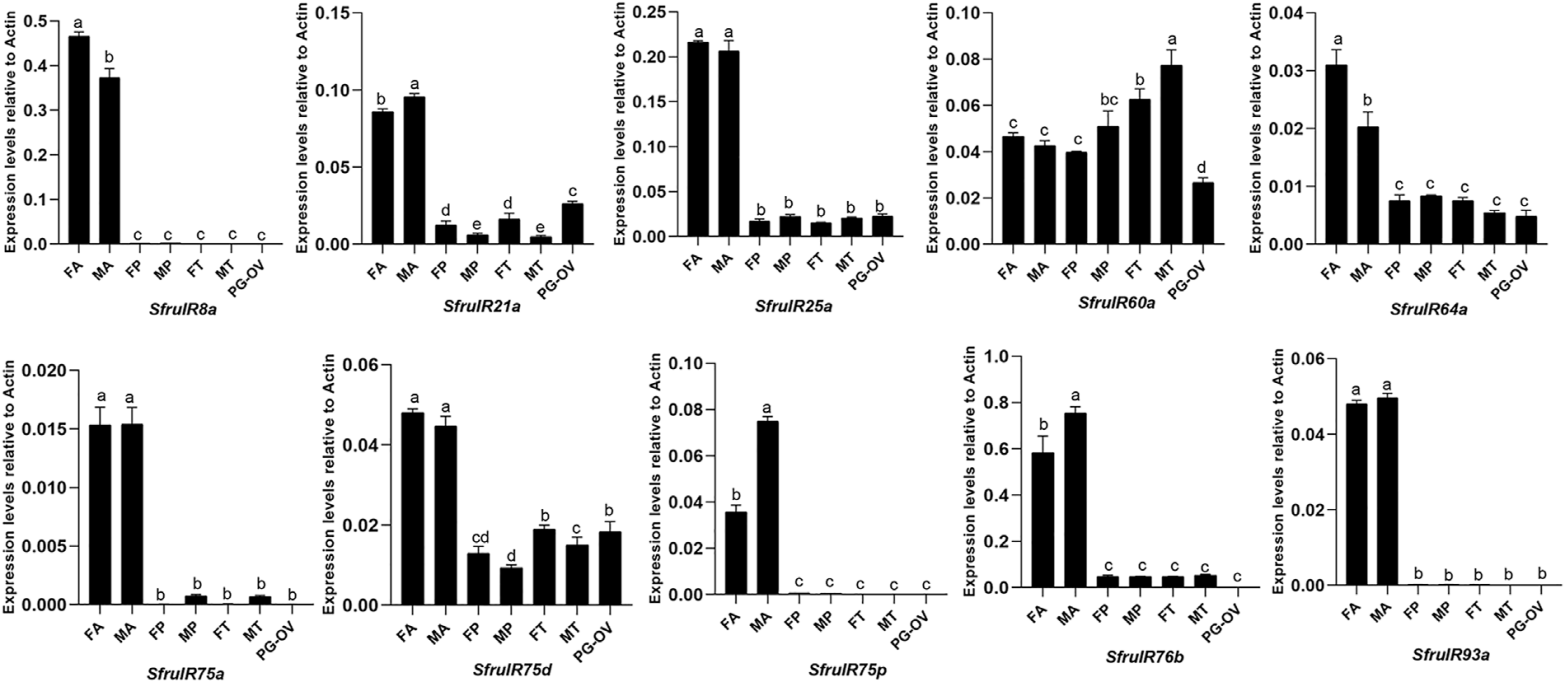
Expression patterns of candidate *SfruIRs* in different chemosensory tissues of adult *S*. *frugiperda*. FA: female antennae, MA: male antennae, FP: female proboscises, MP: male proboscises, FT: female tarsi, MT: male tarsi, PG-OV: female pheromone gland-ovipositor. Different letters indicate significant difference based on a one-way ANOVA followed by Tukey’s multiple comparison test. Error bars show the standard errors of the means (+SE), *p* < 0.05, n = 3.

According to the expression profiles of *SfruIRs* in different chemosensory tissues, 9 of the annotated *SfruIR* genes were mainly expressed in the antennae, especially *SfruIR8a*, *SfruIR75a*, *SfruIR75p*, and *SfruIR93a*, were almost exclusively expressed in the antennae of both sexes. In comparison, the expression of *SfruIR60a*, *SfruIR64a* and *SfruIR75d* was detected in all of the tested tissues. Notably, the expression level of *SfruIR60a* was higher in the tarsi than in other tissues. Moreover, although the expression levels of *SfruIR64a* and *SfruIR75d* were much lower in the tarsi than in the antennae, they were similarly (*SfruIR64a*) or even higher (*SfruIR75d*) expressed in the tarsi comparing to that in the other tissues (Figure 9).

## Discussion

Insect tarsi are important for the feeding behaviors. *S*. *frugiperda* is a worldwide pest which has potential to cause severe damage to more than 300 crop species. Molecular mechanisms underlying the chemosensation of *S*. *frugiperda* tarsi are still unknown. In the current study, we identified 23 ORs, 10 GRs, and 10 IRs in the tarsal transcriptome of *S*. *frugiperda*. This is more than that reported in the tarsi of *H*. *zea* (18 ORs, 9 GRs, and 7 IRs) (Dou et al., 2019), which provides high confidence in the quality of the transcriptome sequencing.

ORs are the vital factor in peripheral olfactory reception in insects (Leal, 2013). A total of 23 SfruORs were identified in our research. Notably, a trace of ORco was documented in the tarsi of *S*. *frugiperda*. Such expression profile may reflect the olfaction roles of the *S*. *frugiperda* tarsi. Furthermore, *SfruORco* was demonstrated to have the highest expression level in the antennae among all the annotated *SfruORs*. This is consistent with the previous suggestion that ORco is the most highly expressed ORs in antennae (Jones et al., 2005; Sun et al., 2020). The *SfruORs* that mainly expressed in the antennae may be involved in the olfaction of *S*. *frugiperda*. For example, HarmOR29, an ortholog of SfruOR62 (identity: 85.44%), mainly expressed in the antennae of *H*. *armigera*, was found to be triggered by methyl 4-hydroxybenzoate when heterologously expressed in *Drosophila* empty neurons (Guo et al., 2021). Although most of the candidate *SfruORs* are extensively expressed in the antennae, *SfruOR30* and *SfruOR53* was also found to be expressed in the tarsi or ovipositor (with lower level than in the antennae). We suggested that these 2 ORs may participate in the detection of feeding- or oviposition-related chemicals (Yactayo-Chang et al., 2021). According to the expression profile analyses, we did not observe obvious expression of other 21 *SfruORs* in the tarsi, this could be explained by the fact that OR genes are mainly expressed in the olfactory organ (i.e., antennae), rather than in the gustatory organs (e.g., tarsi).

In this study, we identified 10 GRs in the tarsi of *S*. *frugiperda*. By sequence alignment and phylogenetic analysis, these GRs were determined in the “CO_2_-receptors” sub-family (SfruGR1/2/3) and “sugar-receptors” sub-family (SfruGR4/5/6/7/8/9/10). We did not annotate putative members in the “bitter-taste receptors” clade. Similarly, only 1 bitter-taste receptor was reported in the tarsal transcriptome of *H*. *zea*. This may be due to the low transcript levels of most bitter-taste receptors (Xu et al., 2016; Yang et al., 2021). And the third-generation sequencing technique (full-length transcriptome sequencing) could help us to identify more receptors of which transcript levels are relatively low in the tested tissues. Furthermore, as shown in the RT-qPCR, in addition to be expressed in the tarsi, most of the annotated *SfruGRs* were also highly expressed in the proboscises. It has long been reported that many chemosensilla are distributed on the mouthpart and that the mouthpart functions in chemosensation in moths (Krenn, 2010; Liu et al., 2014; Hu et al., 2021). Therefore, systematic analysis of GRs in other taste organs such as proboscis are still needed in the future.

Phytophagous insects could detect and measure the CO_2_ gradients so as to direct their oviposition and feeding behaviors. Fresh flowers which produce more CO_2_ than old flowers indicate more nectars than the old ones (Guerenstein and Hildebrand, 2008). It is demonstrated that specialized CSNs that sense CO_2_ are distributed on the labial palps in Lepidoptera (Bogner et al., 1986). And 3 GRs (GR1, GR2, and GR3) are responsible for the detection of CO_2_ (Xu and Anderson, 2015; Ning et al., 2016). In this study, we annotated three CO_2_ GRs (SfruGR1/2/3) in the tarsi of *S*. *frugiperda*. Although moth GRs detecting CO_2_ have been only characterized in antennae and labial palps (Xu and Anderson, 2015; Ning et al., 2016). The identification of three candidate CO_2_ receptors in *S*. *frugiperda* tarsi indicate that moths may also sense CO_2_ *via* their tarsi.

The group of “sugar receptors” in moth GR family is mainly responsible for the detection of various sweet substances. In the transcriptome of *S*. *frugiperda* tarsi, we determined 7 GRs in the clade of sugar-taste receptors. This number is close to that reported in the genome (10 putative sugar-taste receptors) of this species (Gouin et al., 2017). And more than that reported (5) in the tarsal transcriptome of *H*. *zea* (Dou et al., 2019)*.* Although a lot of studies have been made in unrevealing the function of insect sugar-taste GRs in taste perception, most of them have been carried out in the model insect *D*. *melanogaster* (Dahanukar et al., 2007; Moon et al., 2009; Lee et al., 2010; Miyamoto et al., 2012; Dweck and Carlson, 2020). An exception, GR9 homologs in the fructose-receptor clade have been well studied in moths. For example, through heterologous expression, GR9 in *B*. *mori* (BmorGR9) has been demonstrated to be sensitive to *D*-fructose (Sato et al., 2011); GR9 in *H*. *armigera* (HarmGR9) shows responses to *D*-fructose, *D*-galactose, and *D*-maltose (Xu et al., 2016); While in another study, HarmGR9 responds specifically to *D*-fructose (Jiang et al., 2015). In *Spodoptera litura*, SlitGR8 (ortholog of BmorGR9 and HarmGR9) has a specific response to *D*-fructose (Liu et al., 2019). In our study, according to the TPM values and RT-qPCR results, *SfruGR9* was predominantly expressed in the tarsi (with higher levels than the other *SfruGRs*), and the level in female tarsi was significantly higher than that in the male ones, suggesting that SfruGR9 is responsible for the fructose detection during feeding and ovipostion in *S*. *frugiperda*.

Insects IRs not only contribute to olfaction and gustation, but are also involved in the sensing of temperature, humidity, and sound (Rytz et al., 2013; Knecht et al., 2016; Ni et al., 2016; Pitts et al., 2017; Hou et al., 2022). In this study, we annotated a total of 10 IRs in the tarsi of *S*. *frugiperda*. And four of them (SfruIR8a, SfruIR25a, SfruIR76b, and SfruIR93a) were determined in the IR co-receptors clade. RT-qPCR results showed that *SfruIR76b* was expressed with higher levels in the tarsi than that of the other 3 annotated IR co-receptors. It had been demonstrated that IR76b can function as the co-receptor of specific IRs tuned to amino acids in *D*. *melanogaster* (Ganguly et al., 2017). The participation of SfruIR76b in the sensing of amino acids in *S*. *frugiperda* tarsi remains to be elucidated. According to the phylogenetic analysis, the other 6 SfruIRs (SfruIR21a/60a/64a/75a/75d/75p) were grouped in the “antennal IRs”. Consistent with that reported in other insect species (Liu et al., 2018; Zhu et al., 2018), most of the annotated “antennal IRs” were mainly expressed in the antennae of *S*. *frugiperda*. However, *SfruIR60a* was highly expressed in all the tested tissues, and with its levels even higher in the tarsi than in other tissues. Similarly, expression investigation of *HarmIR60a* in olfactory and taste tissues of *H*. *armigera* demonstrated that *HarmIR60a* is expressed in antennae, proboscises, and legs (Liu et al., 2018). It had been found that IR60a group shared higher sequence similarities with divergent-IRs compared to antennal-IRs (Olivier et al., 2011). In our study, SfruIR60a is present between antennal-IRs and divergent-IRs, which is consistent with that reported in other moths (Liu et al., 2018; Hou et al., 2022). Thus, we speculated that SfruIR60a may bear dual roles of taste and smell in the tarsi of *S*. *frugiperda*. Although “divergent IRs” were found to be the largest group in *D*. *melanogaster* (Croset et al., 2010), In the current study, we did not identify members that belonged to the “divergent IRs” in the tarsal transcriptome of *S*. *frugiperda*. This may be caused by low expression levels of “divergent IRs” in the tarsi of *S*. *frugiperda* which need to be experimentally verified in the future.

By analyzing the transcriptome of the tarsi of *S*. *frugiperda*, we annotated 43 putative chemosensory receptor genes. We then used RT-qPCR to compare the expression of these genes in different chemosensory organs. The high expression of several chemosensory receptors in the tarsi suggests that these genes could be important in the tarsal chemosensation of food chemicals in *S*. *frugiperda*. The results should facilitate the study of the molecular mechanisms of chemosensation in the tarsi of *S*. *frugiperda* and of other moth species.

## Data Availability

The datasets presented in this study can be found in online repositories. The names of the repository/repositories and accession number(s) can be found in the article/Supplementary Material.

## Supplementary material

SUPPLEMENTARY FIGURE S1 Homologous species distribution in Nr database.

SUPPLEMENTARY FIGURE S2 Functional annotation of the transcripts based on GO categorization.
