# Supplementary material for: Identification and expression analysis of chemosensory receptors in the tarsi of fall armyworm, Spodoptera frugiperda (J. E. Smith)
Source: Front Physiol. 2023 Apr 10;14:1177297. doi: 10.3389/fphys.2023.1177297 (PMC10123274; doi:10.3389/fphys.2023.1177297)
Supplement: Supplementary file 1 [file DataSheet1.ZIP › Supplementary data/Table S2 (primer).docx]

**Table S2**. Primers for RT-qPCR of candidate *OR*s, *GRs*, and *IRs* in *S*. *frugiperda.*

| **ID** | **Forward primer (5'to 3')** | **Reverse primer (5' to 3')** |
| --- | --- | --- |
| ***ORs*** |  |  |
| *SfruORco* | ATGTTGTCAGATTGGTAGCA | TCTTCAATGAGCCTGTTCC |
| *SfruOR1* | TCCTCCTGTCTTGTTTAGC | GTCCTCATCCCGTTGTAT |
| *SfruOR12b* | AGTAGTTGTAATAGTGGGTCT | GTCTTGATGCCATTTGAT |
| *SfruOR17* | TTCTGGGCAGGCATTTCTT | CAGCGTCTCGAAGTTCATCC |
| *SfruOR25* | ATTACTTATGGGTTTGGG | ACAGGACGTGTATGAGATG |
| *SfruOR27* | CAGTGACCGCAAATCCAA | ATGTTCCGCATTACAGCA |
| *SfruOR30* | CGGTTTCGCAAGCGTCCTA | GCCGCAAGTTATTTGTGAGC |
| *SfruOR32* | CTACACCTCGTATGTGCC | CCCTTCAGAGTTATTTCG |
| *SfruOR34* | CTGTTCGTCCTGTTTCCT | CATATCCTCTTCGGCTTG |
| *SfruOR35* | GCCCTTTGACCCTTACCA | AGAACAGTCTCCGCACCC |
| *SfruOR38* | AACGGAGTGCTGTATGTG | GTAGCCAGTGAGGACGAT |
| *SfruOR40* | AGACGCTGTATTGTGGAG | AGTTCTCGGTATGGTCAA |
| *SfruOR45* | TCAGTTCCATCGCCATCT | CCTCAAACGCCTTATTCC |
| *SfruOR49a* | TGATAATGTCGGGCAACG | TGGCGAATGGAACTGGAA |
| *SfruOR49b* | GAGTACATTAAGAGGAATCGGAAGG | CATAGGCACTGACAATATGACCC |
| *SfruOR50* | TACGCTGCTCAAGGTTTCGG | TGAGGCAGGTGGTCAGGGAG |
| *SfruOR53* | CGCCCACCATGTTTGTTTAT | ATCACGAATCCGTTGCTCTT |
| *SfruOR57* | TCAACCAGACGACGATGAAG | CGTAACGACAGACAGGCAAC |
| *SfruOR62* | TCTTGCTATGGCTCAGTTGG | AACGCTTCTTGAATAAGTTTGC |
| *SfruOR64* | CCTGCTGAGTACCCTGACGC | GAGGAACGCTGTGATGTTGG |
| *SfruOR67a* | CCTTCTTCGCATCCGTTTAT | ATCTGGTCCAGGCTCACATC |
| *SfruOR67c* | CTTACAATGACGCTGCAAAT | TCTTCAACCCTGAGATGACC |
| *SfruOR85c* | CTGAGATGTGGAGGTTGGCTAG | TTTGCTTGGATTCGCTTGTG |
| ***GRs*** |  |  |
| *SfruGR1* | GGTAATCTTCTTCACGACTAC | TCCGAGGTTCACATTCAG |
| *SfruGR2* | GTATCATTATCCGCTCATATCC | ATCCACGATCAGTTCTACAG |
| *SfruGR3* | GGAGTGCTGCCCATTACG | GAACTTGCCTTCCGCTGT |
| *SfruGR4* | TTTCTCCCAATACTAAACAACG | GCTCCTGACATCCGACCT |
| *SfruGR5* | TGCGACAAGTTGACAAGA | CACGACGAGAAACAGGAA |
| *SfruGR6* | CCCGTGCTGTATGATGTG | GCGTCGGAGTAGAGGAGT |
| *SfruGR7* | ATAGCCCTCAACCACATC | AACATCATCCGTTCCTTT |
| *SfruGR8* | CTTCTTAGACGCAATGGA | GCTGTTATGCTTCGGTGT |
| *SfruGR9* | AGGCGGACAATACTCTTTA | AGTACGACGAACCACAGC |
| *SfruGR10* | TTAAGGGAGGACTACACTC | TATTCTCGTATGCTGGAC |
| ***IRs*** |  |  |
| *SfruIR8a* | GACCGCACCCTATACGAG | GTGAGCACCAGGTTCCAG |
| *SfruIR21a* | AGGTTACGCTGGTCACAG | ATTAGGCTCACGAGGTTC |
| *SfruIR25a* | ATAAGGGACGAGCAAGCA | TCAGCCATGACAGACAGC |
| *SfruIR60a* | GTTATCGCCGTACCTCAA | TCCATTACACAGCCATTCA |
| *SfruIR64a* | GAATGGCGTAAGCAATATGT | GAGGAAGGAATAGCGTTAGT |
| *SfruIR75a* | GCCCAGGATAGTGAACCA | TCACCATGCCATCGAAAC |
| *SfruIR75d* | GGACACCACCTACAAGAC | TGATTATGTCGTAGCCAGAG |
| *SfruIR75p* | TCATCATAGGAGCAGTTCTG | AGTGGTGAGTTGAGGAGAT |
| *SfruIR76b* | TGCGGAACAGTGATCTTA | AAACAGCGAGTCGTATGG |
| *SfruIR93a* | ATGGATTCAGAGGGAAGG | GCATCATATCAGTGGTCGT |
| *SfruActin* | TACTCCTAAGCCTGTTGATG | TTATGTCATGGTGCCGAAT |
