# Supplementary figures and images for: Identification and expression analysis of chemosensory receptors in the tarsi of fall armyworm, Spodoptera frugiperda (J. E. Smith)
Source: Front Physiol. 2023 Apr 10;14:1177297. doi: 10.3389/fphys.2023.1177297 (PMC10123274; doi:10.3389/fphys.2023.1177297)

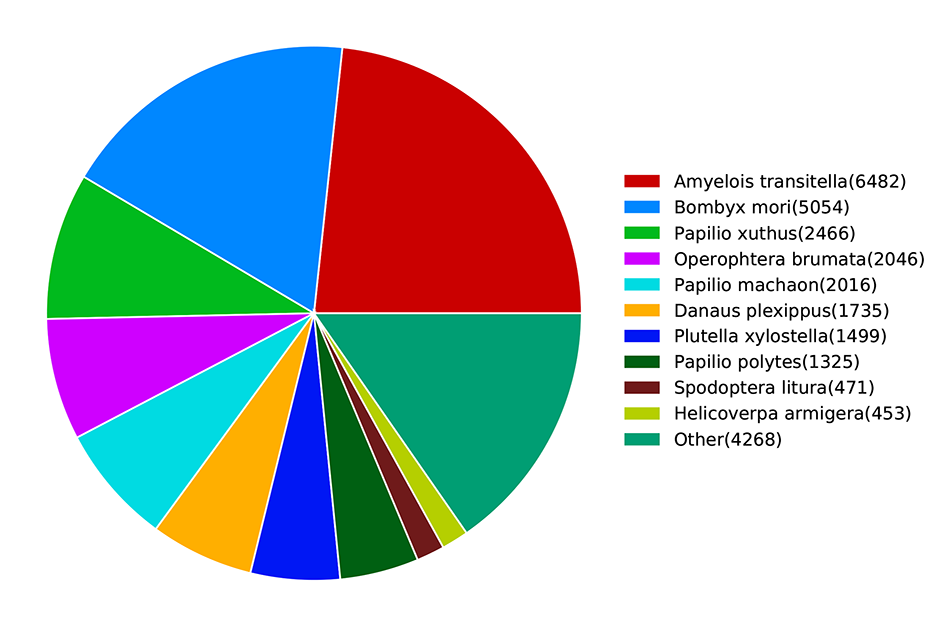

Supplement: Supplementary file 1 [file DataSheet1.ZIP › Supplementary data/Fig S1 (NR).tif]

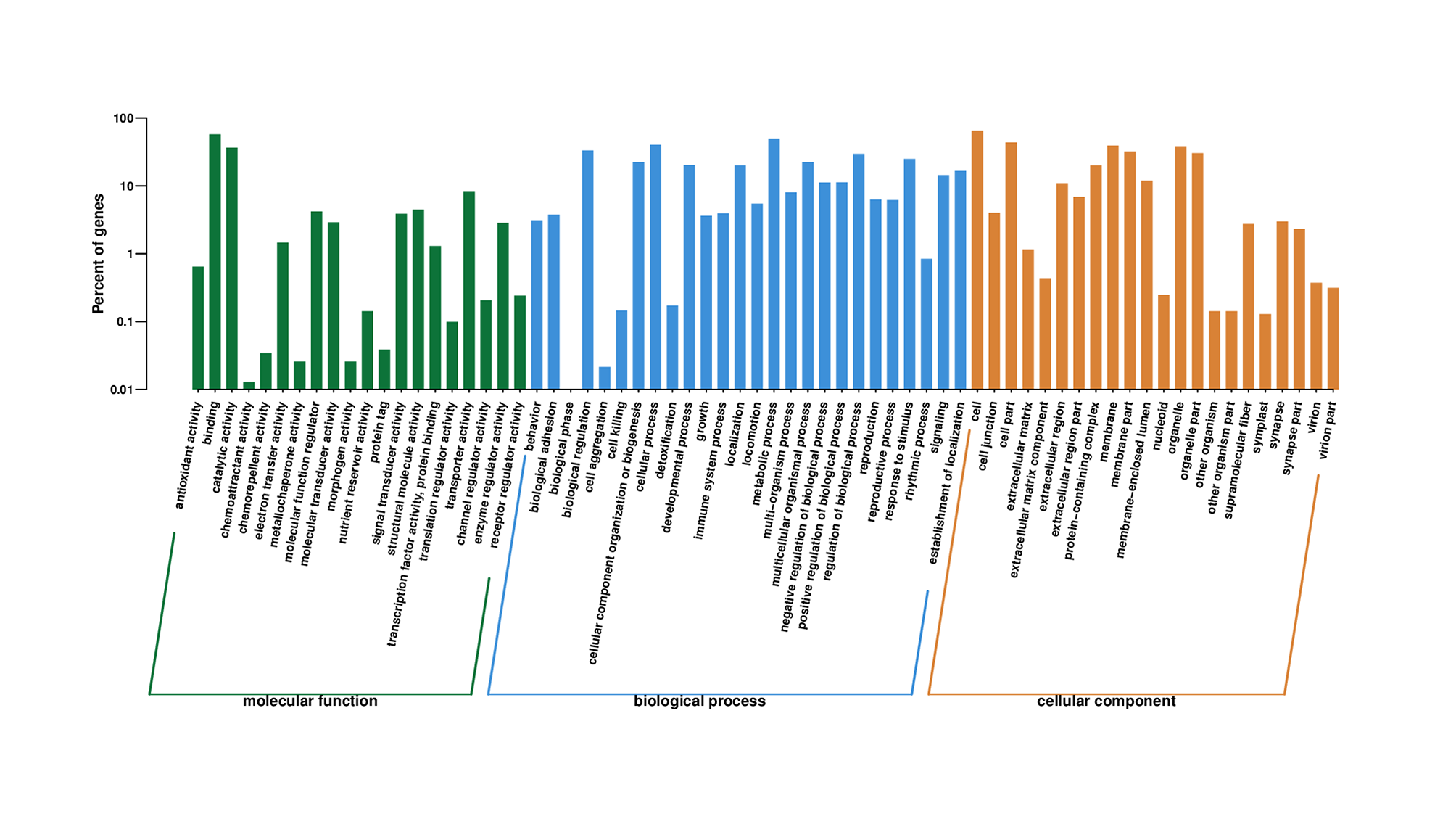

Supplement: Supplementary file 1 [file DataSheet1.ZIP › Supplementary data/Fig S2 (GO).tif]
